# The Tincture of Aconite for Tetanus

**Published:** 1894-12

**Authors:** 


THE TINCTURE OF ACONITE FOR TETANUS.—According to the *Journal de Medecine de Paris*, for May 6, 1894, Wedeman has obtained excellent results from the use of this drug in tetanus, using the tincture in the dose of 5 drops every two hours and afterwards every four hours, or administering the following prescription:

| | | |
|---|---|---|
| ℞. | Chloral hydrate | gr. xxx. |
| | Bromide of potassium | dr. i. |
| | Tincture of opium | drs. ss. |
| | Tincture of aconite | dr. i. |
| | Water | ozs. iii. |

Teaspoonful doses of this mixture may be given every hour.

